# Some Key-notes

**Published:** 1881-09

**Authors:** C. Carleton Smith

**Affiliations:** Philadelphia


### SOME KEY-NOTES.

C. Carleton Smith, M. D., Philadelphia.

Ever since Dr. Guernsey called the attention of the profession to his system of Key-notes, it has been badly abused.

In making prescriptions in homœopathic practice, there is no short cut by which we can jump at once to the proper remedy in a given case. We only become sharp-shooters through hard study, and the more we thumb our materia medica, the greater will be our success and our glory.

If the Key-notes are looked upon by us in the light of guide-posts, pointing us to the *group* of remedies, among which we are to find, by close comparisons, *the remedy* in each particular case of illness, then are they indispensable, and become at once, if thus rightly used, of incalculable value. But if, on the other hand, we attempt to base our prescriptions simply upon these Key-notes as such, we will most assuredly find them stumbling-blocks instead of helps. Let us be careful, therefore, that we do not misinterpret the true meaning and intent of this system.

It is my purpose to call attention to some Key-notes which I have been collecting for some years, and many of which I think are not familiarly known.

Aconite.—Everything tastes bitter except *water*.

Actæa Rac.—Children wake suddenly at night *terrified* and *trembling*, covered with *cool, clammy sweat*.

Æsculus Hip.—Pains *shoot up the rectum* (Ign.) from the hæmorrhoidal tumors, with lameness of the back and aching.

Alœ Socot.—Hard stools drop out without causing the least sensation.

Alumina.—Disposition to *grasp* the seat of the water-closet tightly while at stool. Perspiration breaks out, and the patient despairs of having a stool.

Antimon. Crud.—Child cannot bear to be washed in *cold* water; while under *Sulphur*, the child cannot bear to be washed at all.

Antim. Tart.—*Deathly nausea* relieved by gaseous eructations.

Note.—Children who are not rapidly impressed with this drug in coughs, are by *Hepar S.* In the latter drug we have *purring* and *wheezing*.

Apis Mel.—Can scarcely retain the urine a moment, and, when *passed, scalds* severely. Feels as if he could not take another breath

ARNICA RAD.—Child wants *water* right after nursing, which it always throws up. Eructations bitter, and like rotten eggs. In constipation, when there is violent burning down the back, when the rectum becomes loaded and fæces won't come away. Hæmorrhage from the womb in facial erysipelas, relieving the latter. In pregnancy, fœtus feels as if it laid crossways. *Deathly* coldness in *fore-arms* of children in *Hydrocephalus.*

ARSENIC.—Pain in half of head like a partition from *left fore-head* to left occiput: worse at midnight. The pulse of *Ars.* is *more* rapid in the morning than in the evening. (See *Sulphur.*)

BAPTISIA.—In tuberculosis, chill every *morning* at 11 o'clock and *fever* each afternoon.

BARYTA C.—Suffocating breathing from enlarged tonsils on lying down.

BELLAD.—Flicking before the eyes, with nausea, worse on stooping. *Wets* the *bed* after eating sugar or sweet things. Cough causes acute pain in *left* hypochondria, shooting upwards, worse *lying* on either side or *walking* much. Throbbing in sacrum; has to have pillow stuffed in small of back in order to sit in chair.

BENZOIC ACID. — *Watery* stools, running *right through* the diaper.

BERBERIS.—*Cutting* pain in *left side* of region of bladder, extending into urethra. Bubbling sensation in *kidneys.*

BORAX.—Sensation of *distension* and *stiching* in clitoris at night.

BOVISTA.—Metrorrhagia in *evening* on *lying* down. Menses only at *night* (also *Magn. carb.*).

BROMIUM.—Feeling as if breathing through a *sponge* in the throat. Sharp pain extending into *right* ear from throat in swallowing. (*Kali. B.*, left side.)

BRYONIA.—Neuralgic pain, left side of head and face, relieved by *hard* pressure and cold applications. Water tastes *bitter* (opp. of *Acon.*) Swallowing liquids or saliva more painful than solids. Hiccough *after eating.* (*Nux. V.* from eating *too much* or from *cold drinks.*) *Tightness* above mid-sternum. Swellings of a pale-red blush, with *heaviness* and *hardness.*

CACTUS GRAND.—Discharge of *pure blood* from rectum. Cactus heart-pains come on slowly, increase up to a certain point, and then as gradually subside. (See, also, *Platina.*)

NOTE.—Constriction is the key-note of *Cactus. Arn., Bufo, Iod., Lil. T.* and *Nux mos.*, all have constriction in or about the heart; but *Cactus* is the only drug that has the feeling of a *hand of iron grasping the heart.*

CALADIUM.—Pruritis vulvæ during *pregnancy* and after *miscarriage.*

CALCAREA C.—Flashes of light shoot up from the eyes, then break and fall down in a shower of sparks. She feels better in every way when she is constipated. Strangury brought on by standing on cold damp pavement.

CARBO ANIMAL.—Headache at night; has to sit and hold head with both hands to prevent it from falling to pieces. Deafness; she can hear human voices in the room with her, but cannot tell from whence the sound comes.

CARBO VEG.—Insatiable thirst for cold water; gazing longingly at the empty tumbler, when it is removed from her lips, and asks for more. After-pains felt only in the shin-bones.

CAUSTICUM.—*Peevish* just before the menses (*Cham.*). Larynx feels very *stiff*. Inveterate constipation; stools thin and long-drawn out.

CEPA.—Tickling in larynx is temporarily relieved by eating a piece of apple.

CHAM.—Pressure in the head from within; outward as if top of head would *fly off* or be *blown* off.

CHELIDONIUM.—Excessive lachrymation in orbital neuralgia; the tears fairly gush out, and eyes cannot bear the least light. Flying out of detached lumps of mucus on coughing; the cough reechoes in the stomach.

CINA.—*Enuresis*, with profuse discharge of strong ammoniacal urine. The child is afraid to speak or move for fear she will bring on a paroxysm of cough.

NOTE.—*Silicea* follows well in vermiculous subjects when *Cina* fails.

CHINA.—In icterus, when there is great *depression*, feebleness and *breathlessness.*

QUININE.—Can only see objects by looking sideways.

COCCULUS.—Umbilical hernia if *Nux* fails and there is stubborn constipation.

COLCHICUM.—Nose, color of bleached wood-ashes. Tongue covered with a *downy* white fur. Nausea in the sick from odor of cooking food.

COLOCYNTH.—Aggravation from *cheese.*

CROCUS.—Thumping and knocking throughout the brain.

CUPRUM MET.—Cough sounds as if water was being poured from a bottle.

Cuprum Acet.—Constant protrusion and retraction of the tongue like a snake. In epilepsy, aura begins at knees, ascending until it reaches the hypogastric region, when unconsciousness occurs, foam at the mouth, and falling down convulsed. Soon as patient goes into a high ceiling room, the head reels and she loses her senses.

Cyclamen Europ.—After confinement, patient has colicky bearing-down pains, each pain accompanied by a gush of blood, which relieves the pain momentarily.

Aranea Diadema.—She awakens at night with hands feeling twice their natural size, so that she cannot make any use of them.

Digitalis.—Shuddering in the mammæ; feels as if heart would stop beating if she dared to move. (*Gels.* just the opposite.)

Dulcamara.—Catarrhal ischuria in grown-up children from wading with bare feet in water, with discharge of mucus and milky urine.

Euphrasia.—Cough always excited if exposed to *south wind.*

Gelsemium.—In "ague," patient wants to be *held* during the shakes; sleeps throughout the heat; thirst *during sweat;* muttering delirium when half awake.

Graphites.—Feeling of cobweb on right forehead; tries hard to brush it off. Hearing is improved in deaf people while riding in a carriage; hears better when in a *noise.*

Hepar Sulph.—Feels as if eye-balls were *drawn back into head.* (Also, *Asterias*, *Bovista*, *Cham.*, *Plumb.*, *Rhod.*, *Sil.*, *Strychnia*, *Sulph.*, *Paris Quad.*) Looking at an object steadily makes eyes water.

Ignatia.—Trembling of hands; worse right side when writing, and when being watched; worse, also, from extending the fingers.

Iodium.—Child very cross, cannot bear to be looked at or touched (See *Ant. Tart.*) *Itching* in the lungs low down, and extending upward through trachea to nasal cavity. The itching in end of nose is the signal for the cough to begin. Sensation as if heart were *squeezed* (*Cactus*).

Jatropha.—Great dizziness, with constant nausea; better in open air.

Kali Brom.—He imagines he is especially singled out as an object of Divine vengeance. Thinks all her friends have deserted her. Extreme *drowsiness*; constant hacking cough proceeding from the *chest*, during pregnancy. Irresistible desire to urinate, but no flow except after great urging, and then with difficulty.

Kali Carb.—Sensation as if a stick extended from throat to left side of abdomen with a ball on each end of stick. Belching of putrid gas like rotten eggs. Stomach feels as if it would surely burst. Hard, white, round masses fly from mouth when coughing or hawking. (*Chelidon.*) Heart feels as if suspended from left rib.

Lachesis.—The least movement causes feeling of suffocation around the heart. Intolerable pinching and itching in spots on lower extremities relieved only by plunging in *cold* water; worse after sleep. The tenesmus caused by this drug is relieved by *Sepia.*

Lycopodium.—Sensation as if *hot* balls dropped from each breast through to back, running down back, along each leg to heels and dropping off at heels. This sensation alternating with feeling as if balls of ice followed the same course. The fœtus seems to be constantly turning somersaults within the womb.

Note.—Especially useful in dry cough, day and night, in feeble, emaciated boys. Chill every seventh day.

Mag. Mur.—Stools knotty like sheep's dung, crumbling at the verge of the anus and covered with mucus and blood. Urine scanty, can only be passed by bearing down hard with the abdominal muscles.

Note.—Always to be studied in uterine diseases connected with *hysterical* complaints. Tendency of head to sweat (like *Calc*). Has headache (like *Silicea*). Better from wrapping up the head, but unlike *Silicea* worse in open air. Inability in children to digest milk, it always causes pain in stomach and passes undigested.

Metrorrhagia, always worse at night in bed, accompanied with spasms of uterus, causing hysteria. Tendency of feet to sweat (*Silicea*).

Mangan. Acet.—Pain in right ear proceeding from *sound* teeth. Ear so sore cannot lie on that side. Burning of ears as if from very hot stove.

Mercurius Sol.—Greenish painless *gonorrhœa,* especially at night. Agg. from cold air coming in contact with exposed parts; for instance, exposure in out-door water-closets.

Merc. Cor. Sub.—Gonorrhœa at first thin, afterward thick, and at last with *biting* pain on urinating, and stitches extending *back* through the *urethra.*

Muriat. Acid.—All the time keeps pushing his finger down his throat, or keeps *clawing* at his mouth.

Nux Vom.—Child cries and squirms for an hour after eating. Sweats only on right side.

Oleander.—Headache improved by looking either sideways or cross-eyed.

Opium.—Violent movements of the fœtus, especially toward night, preventing sleep.

Oxalic Acid.—*Burning* in throat accompanying abdominal pains.

Petroleum.—Vertigo in *back* of head. Gastralgia when the stomach becomes empty.

Phosphorus.—Vertigo as if a veil obstructed the sight, with inability to *think*. Feels that he will surely fall. Don't know where he is; worse at noon. During pregnancy she cannot drink water; the sight of it causes her to vomit, and she must close her eyes while bathing. Anus remains open all the time; the child strains at stool. Small ulcers clustering around large ones, some healing, and others healed.

Phos. Acid.—Can't get up after sitting, from pain in left hip.

Phytolacca.—The tumefied breast neither heals nor suppurates, is of a purple hue and as hard as old cheese. This drug stands between *Bry.* and *Rhus.*, and cures when these fail, when apparently indicated.

Plumbum Acet.—Sensation of a body rising up to the throat and extending to both ears pressing up into them, causing swallowing which makes it descend, soon to return; worse from 9 A. M. to noon.

Psorinum.—Pain in occiput from right to left as if a piece of wood was laid on back of head. Eructations like *rotten eggs.* Also, *Arn.*, *Tart. Em.* and *Graph.* *Arnica* has taste of rotten eggs, especially in A. M. *Tart. Em.* at night, and *Graph.* only in A. M., after rising, disappearing on rinsing the mouth.

*Psor.*—Has stools smelling like rotten eggs. *Cham.* has stools smelling like *spoiled eggs*, but *not* the *spoiled egg* flutulence or eructations. Under *Psorinum* the soft stool is voided with difficulty, *from weakness.* Normal stool, but passed in a great hurry, can hardly reach the water-closet, with quantities of flatus. Prolapsus recti with *burning* and *sticking.* Must keep the arms spread wide apart in order to breathe freely. Want of breath in the open air, has to hurry home and lie down in order to breathe freely. Weakness in all the joints of the body as if they would not hold together.

Ranunc. Bulb.—Superficial pains upon the external chest of a *sharp, shooting, tearing, stitching* character, coming in paroxysms.

Rheum.—Hunger, but a *mouthful* satisfies.

RHODODENRON,—Has improvement from wrapping head up warmly, like *Silicea.* Speechless and breathless from violent pleuritic pain, running downward in left anterior chest after standing on cold ground. Paroxysmal chorea, *left arm, leg* and *face* on approach of a storm. Cannot get to sleep or remain asleep, unless legs are crossed.

SABADILLA.—Headache better from looking *fixedly* at some object.

SAMBUCUS.—Child *inspires,* but cannot *expire;* face livid.

SARSAPARILLA.—Urine only dribbles while sitting, but when *standing* it passes freely. Feels as if bound down to the bed by a powerful *suction,* with sharp pains in back and shoulders.

SEPIA.—Child coughs till breath is gone, and then gags and vomits mucus. Cough constant when the child is laid down.

STANNUM.—Has colic better from leaning over something hard (like *Coloc.*) In worm fever child lies on abdomen during the pains.

